# Impact of prior cancer history on survival of patients with hypopharyngeal cancer

**DOI:** 10.1002/cam4.5208

**Published:** 2022-09-04

**Authors:** Qi‐Wei Liang, Shu‐Yi Hong, Liang Peng, Jing Liao, Wei‐Ping Wen, Wei Sun

**Affiliations:** ^1^ Department of Otorhinolaryngology Head and Neck Surgery, The First Affiliated Hospital Sun Yat‐sen University Guangzhou China; ^2^ Department of Otorhinolaryngology Head and Neck Surgery Department of Thyroid Center/Thyroid Surgery, The Sixth Affiliated Hospital Sun Yat‐sen University Guangzhou China; ^3^ Department of Otorhinolaryngology of Longgang Center Hospital, the Ninth People's Hospital of Shenzhen Shenzhen China

**Keywords:** hypopharyngeal cancer, prior cancer, survival, SEER, trial eligibility

## Abstract

**Background:**

The impact of prior cancer history on survival of hypopharyngeal cancer patients remains unknown. The present study assessed the impact of prior cancer history on survival of patients with hypopharyngeal cancer.

**Methods:**

Patients with primary hypopharyngeal cancer diagnosed between 2004 and 2015 were extracted from the Surveillance, Epidemiology, and End Results (SEER) database. Propensity score matching (PSM) was conducted to balance baseline characteristics. One‐to‐one PSM, Kaplan–Meier method, and log‐rank test were performed for survival analysis.

**Results:**

We included 5017 patients with hypopharyngeal cancer. Prior cancer history had no significant impact on overall survival of hypopharyngeal cancer patients in comparison with those without prior cancer history (*p* = 0.845, after PSM). Subgroup analysis showed that prior cancer history had no significant effect on overall survival of hypopharyngeal cancer patients.

**Conclusion:**

More hypopharyngeal cancer patients with prior cancer history should be considered for clinical trials. However, further prospective studies are needed.

## Graphical abstract

In this paper, we assessed the impact of prior cancer history on survival of patients with hypopharyngeal cancer. The results showed that the previous cancer diagnoses did not significantly impact the hypopharyngeal carcinoma patients' overall survival, which means maybe more hypopharyngeal cancer patients with prior cancer should be considered for clinical trials.

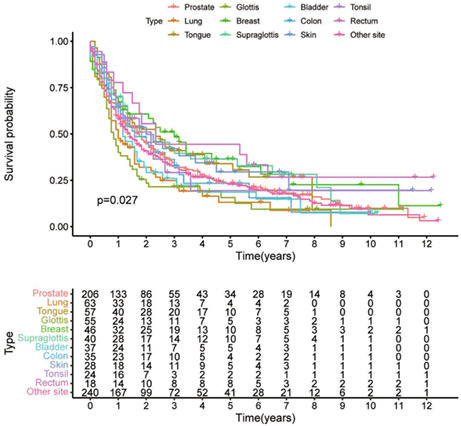

## 1 INTRODUCTION

Many individuals who are diagnosed with incident cancer have already survived previous cancer for >5 years. In the United States, it is anticipated that the number of cancer survivors has increased almost fivefold to approximately 16 million in the past 30 years, which undoubtedly increases the risk of multiple primary cancers.^1^ Unfortunately, most of these patients were not included in clinical trials because of the influence of previous cancer on survival and treatment.^2^, ^3^ However, in a pan‐cancer analysis, the survival of newly diagnosed cancer patients was unaffected by past cancer history.^4^ Several other retrospective studies have reached similar conclusions.^2^, ^5^ Based on current predictions, cancer survivors' clinical trial participation rate is approximately 8%, which leads to a low registration rate and under‐representation, which does not benefit this group of patients. Notably, this exclusion criterion is often adopted without scientific verification.^6^

Hypopharyngeal carcinoma is only the fifth most prevalent malignancy in the head and neck, responsible for 3%–5% of head and neck cancers with roughly 84,200 new diagnoses and 38,600 deaths annually on a global scale.^7^, ^8^ However, the 5‐year survival rate is about 20%, which is lower than cancer of the larynx, oropharynx, or oral cavity.^9^ In recent years, hypopharyngeal cancer has been diagnosed as the second primary tumor in an increasing number of cancer survivors. Clinical studies are necessary to elevate the chance of survival among these patients; however, they are excluded from the majority of clinical research.^10^, ^11^, ^12^ The low incidence and survival rate of hypopharyngeal carcinoma means that the exclusion criteria should be more cautious in order to benefit more patients. However, it is not clear whether previous cancer diagnoses affect the survival outcomes among patients with hypopharyngeal carcinoma. This challenge was addressed by utilizing the Surveillance, Epidemiology, and End Results (SEER) database to evaluate the effect of previous cancer diagnoses on hypopharyngeal carcinoma patients' prognoses to assist with clinical research on these patients.

## 2 METHODS

### 2.1 Data collection

All the information in this research was derived from the SEER database, which contains information on approximately 28 percent of the population of the United States and was accessed with the SEER*Stat program (version: 8.3.6). The patients with confirmed diagnoses of hypopharyngeal squamous cell carcinoma (site code C13.0–13.9) between 2004 and 2015 were retrieved from this repository. Only individuals having a single primary tumor or who previously had a malignant tumor were considered for inclusion. Eligibility conditions for exclusion were as follows: age < 18 years, unknown date of diagnosis or death, or a lack of complete data on survival and follow‐up. The demographic and clinical features were acquired from the SEER repository, encompassing sex, race, age, marital status, year of diagnosis, TNM staging (sixth edition of the American Joint Commission on Cancer [AJCC]), pathological grading, and treatment (surgery, radiotherapy, or chemotherapy). The marital status was classified into married, single, or other status (domestic partner, separated, widowed, or divorced). Finally, we enrolled 5017 patients. The main outcome indicator was overall survival (OS).

### 2.2 Statistical analyses

The patients were categorized into two groups: primary hypopharyngeal carcinoma and second primary hypopharyngeal carcinoma. To eliminate selection deviation and confusion, one‐to‐one propensity score matching (PSM) was employed. The clinical and demographic characteristics of these two groups of patients were evaluated utilizing descriptive statistics. The balance of baseline characteristics was evaluated by absolute standardized deviation (ASD), with ASD <0.1 considered to well balance. The covariates we used for PSM included sex, race, age, marital status, year of diagnosis, TNM staging, pathological grading, and treatment. They need to be balanced because they may affect survival prognosis of patients. The clinicopathological characteristics of the two groups were subjected to a comparison utilizing Pearson's chi‐square test. Subsequently, the Kaplan–Meier technique was utilized to determine the overall survival (OS) rates of the two groups, and the log‐rank test was employed to evaluate the survival curves of patients in the two different groups. Two‐tailed *p* < 0.05 was established as the criterion for statistical significance. The R version 3.6.0 (http://www.r‐project.org/) was adopted to execute all analyses of statistical data.

## 3 RESULTS

### 3.1 Baseline characteristics

We identified 5017 eligible hypopharyngeal carcinoma patients whose diagnoses were made between 2004 and 2015. Eight hundred and eighty‐five of these patients (17.64%) had a prior cancer history. Two hundred and seventy‐nine (31.53%) of the patients had previously been diagnosed with cancer that was classified as AJCC stage I/II. After hypopharyngeal carcinoma diagnosis, the median time interval (IQR) between the hypopharyngeal carcinoma and the previous cancer was 67 (30–110) months, whereas the median follow‐up (IQR) was 16 (7–48) months after the hypopharyngeal carcinoma diagnosis (Table 1). After controlling for PSM, all factors were evenly distributed among patients who had and did not have past malignancy (Table 2). Baseline patient characteristics are displayed in Table 2. Thirty‐six patients with cancer history were excluded in the matched dataset and the information of these 36 patients is shown in Table Table S1. Older adults were more likely than younger adults to have had a past cancer diagnosis (aged ≥65 years, 68.70% vs. 43.80%, *p* < 0.001) and married (51.64% vs. 43.76%, *p* < 0.001) patients. The most prevalent types of previous malignancies included prostate (206, 24.26%), lung (63, 7.42%), tongue (57, 6.71%), and glottis (55, 6.48%) (Table 3). The types of cancers in “other sites” included locomotor system cancer, nervous system cancer, endocrine system cancer, circulatory system cancer, respiratory system cancer, digestive system cancer, urinary system cancer, reproductive system cancer, for example, bones and joints, brain and other nervous system, esophagus, stomach, liver, Hodgkin lymphoma, non‐Hodgkin lymphoma, melanoma, non‐melanoma of skin, myeloma, urinary bladder, thyroid, and so on.

**TABLE 1:** Summary description of demographic and clinical factors of patient with prior cancer(*N* = 885)

	At prior cancer diagnosis	At hypopharyngeal cancer diagnosis
Variable	Value	Value
Age, *n* (%)		
<65	484 (54.69)	278 (31.41)
≥65	401 (45.31)	607 (68.59)
ACC stage, *n* (%)		
I–II	279 (31.53)	178 (20.11)
III–IV	127 (14.35)	558 (63.05)
Unknown	479 (54.12)	149 (16.84)
Interval between diagnoses, months		Follow up from hypopharyngeal of larynx cancer, months
Mean	82.07	28.46
Median (IQR)	67 (30–110)	16 (7–48)

Abbreviation: IQR, interquartile range.

**TABLE 2:** Baseline characteristics of patients with hypopharyngeal cancer

Characteristic	Original dataset					Matched dataset				
	No prior cancer (*N* = 4132)		Prior cancer (*N* = 885)		ASD	No prior cancer (*N* = 849)		Prior cancer (*N* = 849)		ASD
Age					0.062					0.005
<65	2322	56.20	277	31.30		263	30.98	277	32.63	
≥65	1810	43.80	608	68.70		586	69.02	572	67.37	
Year of diagnose					0.076					<0.001
2004–2009	2083	50.41	393.00	44.41		432	50.88	385	45.35	
2010–2015	2049	49.59	492.00	55.59		417	49.12	464	54.65	
Race					0.135					0.006
White	3136	75.90	715	80.79		674	79.39	679	79.98	
Black	718	17.38	122	13.79		114	13.43	122	14.37	
Others/Unknown	278	6.73	48	5.42		61	7.18	48	5.65	
Gender					0.043					0.030
Female	781	18.90	178	20.11		191	22.50	169	19.91	
Male	3351	81.10	707	79.89		658	77.50	680	80.09	
Marital status					0.697					0.074
Single	1020	24.69	135	15.25		172	20.26	133	15.67	
Married	1808	43.76	457	51.64		362	42.64	435	51.24	
Other status	1115	26.98	247	27.91		279	32.86	238	28.03	
Unknown	189	4.57	46	5.20		36	4.24	43	5.06	
Grade					0.380					0.045
Grade I	148	3.58	47	5.31		40	4.71	40	4.71	
Grade II	1544	37.37	352	39.77		307	36.16	331	38.99	
Grade III	1305	31.58	284	32.09		284	33.45	276	32.51	
Grade V	60	1.45	7	0.79		14	1.65	7	0.82	
Unknown	1075	26.02	195	22.03		204	24.03	195	22.97	
AJCC					0.824					0.043
I–II	449	10.87	179	20.23		118	13.90	164	19.32	
III–IV	3278	79.33	557	62.94		651	76.68	539	63.49	
Unknown	405	9.80	149	16.84		80	9.42	146	17.20	
Surgery					0.465					0.062
No/unknown	3453	83.57	624	70.51		605	71.26	624	73.50	
Yes	679	16.43	261	29.49		244	28.74	225	26.50	
Chemotherapy					0.573					0.020
No/unknown	1438	34.80	485	54.80		417	49.12	449	52.89	
Yes	2694	65.20	400	45.20		432	50.88	400	47.11	
Radiotherapy					0.792					0.056
No/unknown	1043	25.24	345	38.98		305	35.92	311	36.63	
Yes	3089	74.76	540	61.02		544	64.08	538	63.37	

Abbreviation: ASD, absolute standardized deviation.

**TABLE 3:** Distributions of prior cancer types

Prior cancer type	Number	Proportion (%)
Other site	240	28.27
Prostate	206	24.26
Lung	63	7.42
Tongue	57	6.71
Glottis	55	6.48
Breast	46	5.42
Supraglottis	40	4.71
Bladder	37	4.36
Colon	35	4.12
Skin	28	3.30
Tonsil	24	2.83
Rectum	18	2.12

### 3.2 Impact of past cancer diagnoses on the OS of individuals with hypopharyngeal carcinoma

**FIGURE 1:**
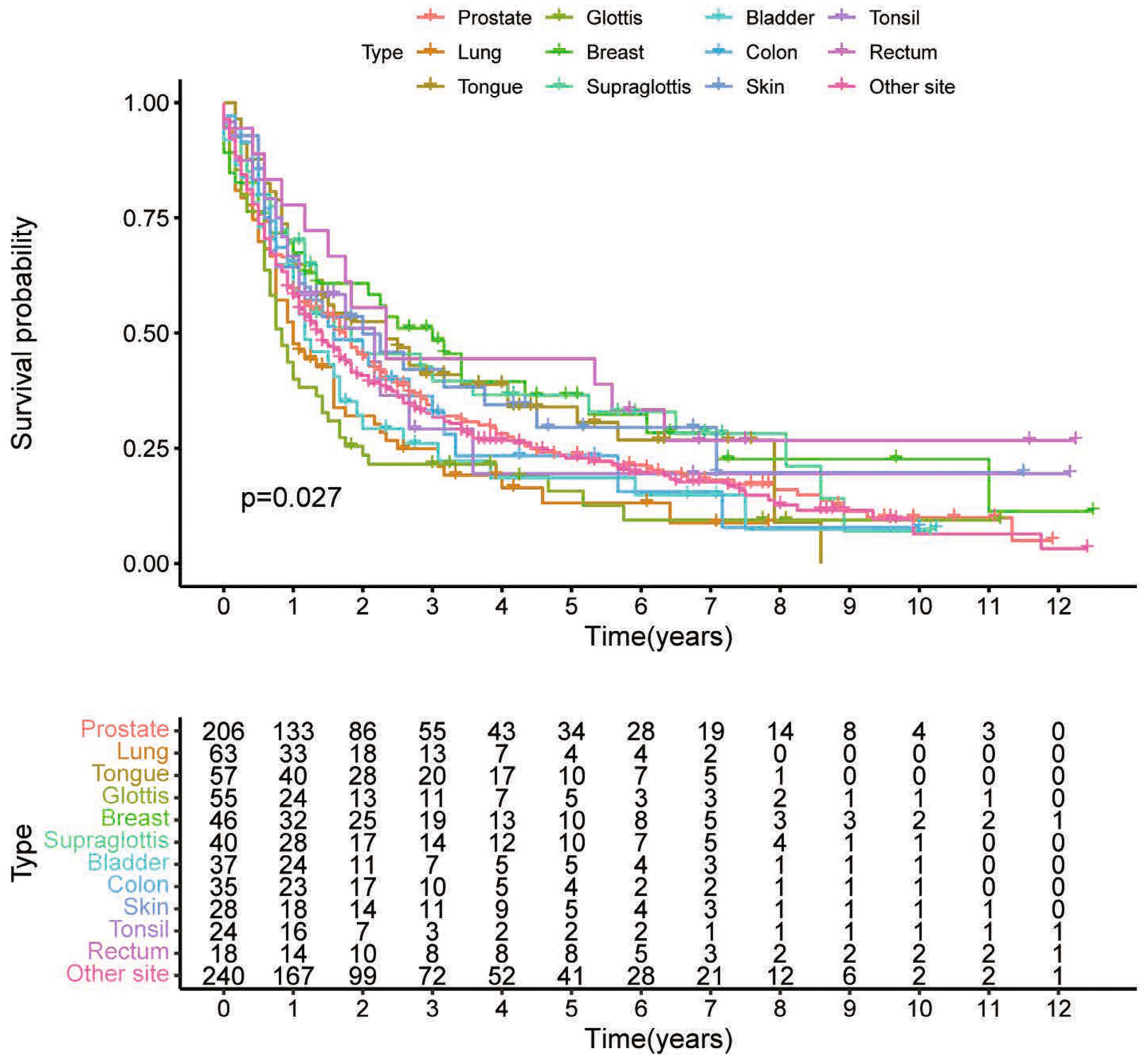
Overall survival (OS) of hypopharyngeal carcinoma patients with distinct forms of past cancers.

**FIGURE 2:**
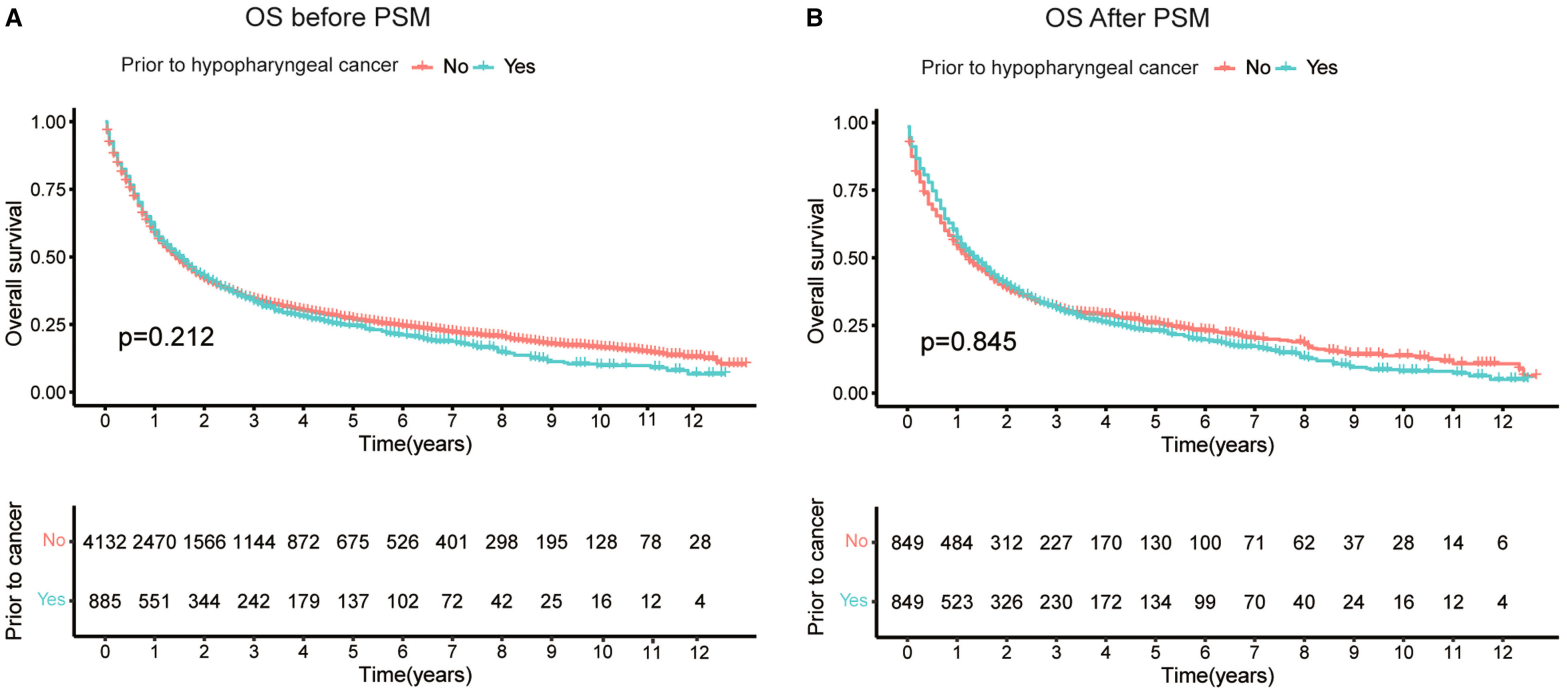
Kaplan–Meier survival curves illustrating the influence of past malignancy on the overall survival (OS) of hypopharyngeal carcinoma patients with or without past cancers. (A) OS analysis prior to conducting propensity score matching (PSM); (B) OS analysis following the PSM.

**FIGURE 3:**
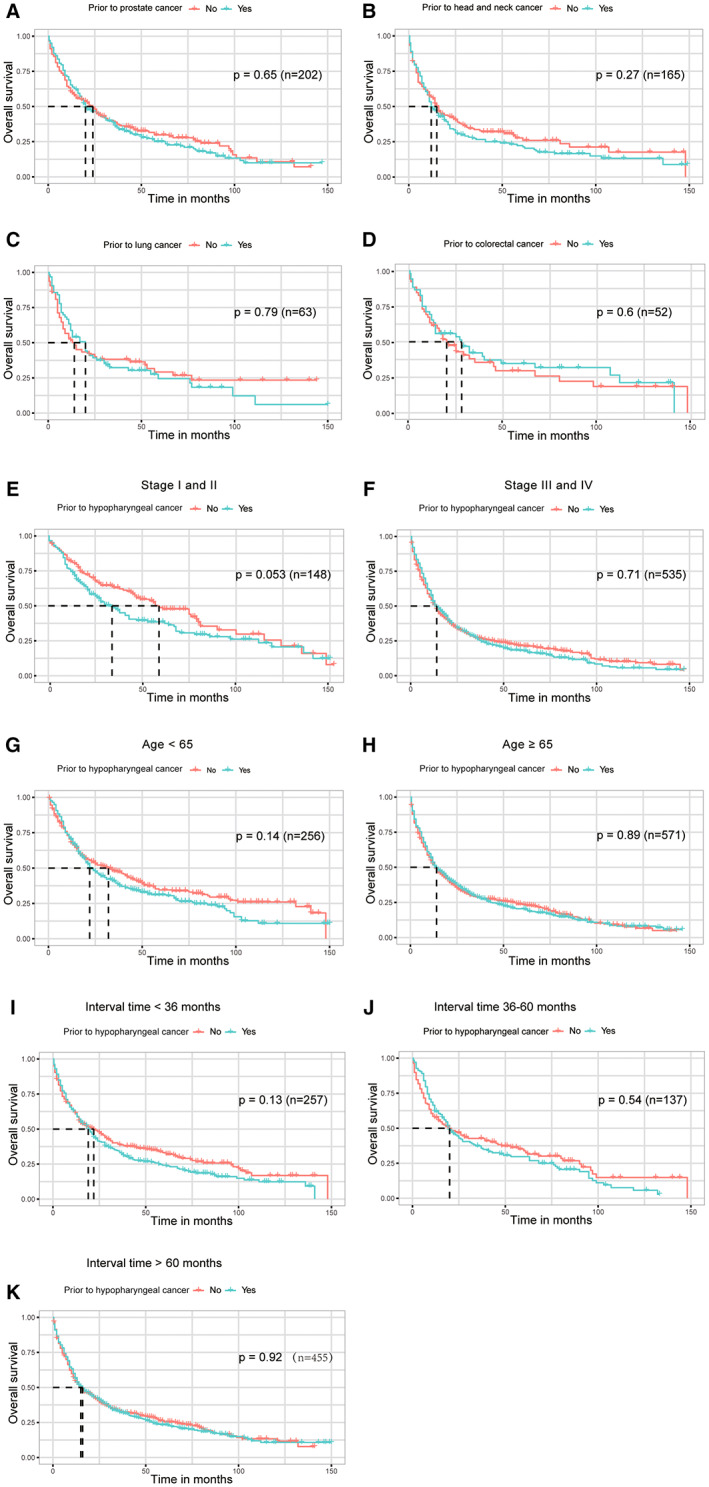
Subgroup study of the impact of past cancer on overall survival (OS) in hypopharyngeal carcinoma patients with or without previous cancer. (A) The influence of previous prostate cancer on OS; (B) The influence of past head and neck cancer on OS; (C) The influence of past lung cancer on overall survival; (D) The influence of previous colorectal cancer on OS; (E) OS for AJCC stage I/II; (F) OS for AJCC stage III/IV; (G) OS for age < 65 years; (H) OS for age ≥ 65 years; (I) Analyses of OS with a < 36‐month timeframe; (J) Analyses of OS with a timeframe of 36 to 60 months; (K) Analyses of OS analysis with timeframe >60 months. Head and neck cancer included tongue, glottis, supraglottis, and tonsil cancer; colorectal cancer included colon and rectum cancer.

On the basis of an adjusted Kaplan–Meier analysis of the matching dataset, the overall survival (OS) of hypopharyngeal carcinoma patients with various forms of previous malignancy is shown in Figure 1. The hypopharyngeal carcinoma patients' prognoses were unaffected by a previous diagnosis of prostate cancer. Additionally, patients having a cancer history had an OS that was comparable to those without a history of cancer (Figure 2A). Figure 2B shows a similar result after PSM (*p* = 0.845). We conducted subgroup analyses among patients classified based on the kinds of past malignancy, AJCC stage, age, and timing. The impact of past cancer on hypopharyngeal carcinoma patients' OS did not differ by form of cancer (Figure 3A–D). This indicated that prior prostate, head, neck, lung, or colorectal cancers did not significantly affect the OS of hypopharyngeal carcinoma patients. Previous malignancy history was not linked to an unfavorable OS among individuals of distinct ages (<65 or ≥ 65 years) or AJCC histological grades (I/II or III/IV) (Figure 3E–H). No substantial differences in survival were found between patients with previous cancer diagnoses and those who did not have a previous cancer history, irrespective of the period between the index hypopharyngeal carcinoma and the previous cancer diagnoses (Figure 3I–K). In the study, it was the OS compared inter groups of patients with and without cancer history. The survival analysis for the subgroup in Figure 3 is all rematched and the baseline data of these subgroups before and after PSM are provided in Table S2–S12.

## 4 DISCUSSION

The results of this investigation could have significance for the exclusion criteria used in contemporary clinical studies. Patients with past confirmed diagnoses of cancer are frequently omitted from clinical trials at present due to the perception that the past disease may have an impact on the survival results. However, rigorous qualifying conditions may prevent the recruitment of many patients into clinical trials, thus impeding these experiments, especially among rare malignancies with low incidence.^13^ Our results showed that previous cancer diagnoses did not significantly impact the hypopharyngeal carcinoma patients' OS, irrespective of the whole group or subgroup analysis. To our knowledge, this is the first research to assess the influence of past cancer diagnosis on hypopharyngeal carcinoma. Earlier research reports have shown that having a past cancer history may not have a negative impact on the patients' OS among those who develop a secondary malignancy, such as lung, nasopharyngeal, esophageal, or gastrointestinal cancer, congruent to the findings of the current study.^4^, ^14^, ^15^, ^16^ In our retrospective study, the SEER database was utilized to obtain data on 5017 cancer patients, where about 1/6 patients (*n* = 885, 17.64%) had a prior cancer history, which is a high incidence compared with other cancers studied.^3^, ^14^, ^17^ This suggests that if prior cancer history is an exclusion criterion, it may affect the registration rate and representation of clinical trials. The median time interval between hypopharyngeal carcinoma diagnosis and the previous cancer was 67 months, which is longer than that previously reported for several other cancers.^18^, ^19^, ^20^ The possible reason is that hypopharyngeal carcinoma is difficult to detect early, and it is often at an advanced stage when diagnosed.^21^ In subgroup analysis, history of prostate, head and neck, lung, or colorectal cancer did not considerably affect the OS of patients with hypopharyngeal carcinoma. Bias tends to occur in other prior cancer types because of insufficient samples, such as breast cancer (*n* = 46, 5.42%). Therefore, these were not included in the comparison. These cancers are heterogeneous illnesses that could share a number of risk factors, such as advancing age, alcohol use, and smoking. In our research, past cancer diagnoses were not linked to the unfavorable OS in patients with different timing, ages (<65 or ≥ 65 years), or AJCC histological grades (I/II or III/IV). However, patients with AJCC I/II stage cancer without a prior cancer history had higher OS, although this was not significant (*p* = 0.053). The lack of significant difference may have resulted from the insufficient sample. In most subgroups, previous cancer diagnoses were found to have no remarkable impact on the OS of hypopharyngeal carcinoma patients. On the basis of our findings, we suggest that more hypopharyngeal carcinoma patients with a previous cancer diagnosis ought to be incorporated in clinical studies, especially considering the low prevalence and survival rate of hypopharyngeal carcinoma. In addition to potential concerns about survival, another reason to exclude this group of patients from a trial is that they may have previously received targeted therapy, radiotherapy, or chemotherapy, which may affect the efficacy of the trial.^2^ However, prior cancer diagnosis as a simple exclusion criterion is an ineffective and excessively broad strategy. In this research, 70% of cancers in patients who registered the staging status were in situ or local stage (AJCC I/II), and they may not have been treated with radiotherapy or chemotherapy. Perhaps, a more appropriate approach would be to exclude previous cancer treatments or limit registration by organ function.

There were a number of drawbacks to the current investigation. First, the retrospective databases had inherent selection bias, although we used PSM to reduce this bias to the extent possible. Second, we only had partial characteristics of prior cancers (without details) because of the constraints of the database. In the present research, we simply took into account the time of the past malignancy and did not take into account any other clinical characteristics or therapies. Third, insufficient samples in the subgroup analysis may have led to biased results on account of the low incidence of hypopharyngeal carcinoma. Therefore, it is necessary to conduct prospective studies in the future.

## 5 CONCLUSION

Except for rare cases, prior cancer has no significant effect on the clinical prognosis of hypopharyngeal carcinoma. We suggest that clinical studies can be considered for hypopharyngeal carcinoma patients with past malignancies temporarily, and more patients can be enrolled in studies. However, further prospective studies are needed.

## AUTHOR CONTRIBUTIONS

**Qiwei Liang:** Conceptualization (equal); formal analysis (lead); writing – original draft (lead). **Shuyi Hong:** Data curation (lead); methodology (equal); writing – original draft (supporting). **Liang Peng:** Software (lead); supervision (lead). **Jing Liao:** Data curation (lead); project administration (lead). **Weiping Wen:** Resources (equal); supervision (equal); validation (supporting); writing – review and editing (supporting). **Wei Sun:** Funding acquisition (lead); resources (equal); supervision (equal); validation (equal); writing – review and editing (lead).

## FUNDING INFORMATION

This work was supported by the National Natural Science Foundation of China, Grant/Award Numbers:81972527.

## ETHICS STATEMENT

This study was approved by the sixth Affiliated Hospital of Sun Yat‐sen University. The study did not require informed patient consent because the data obtained from the SEER database are de‐identified patient data. All analyses conducted in this study were in accordance with the 1964 Helsinki declaration and its later amendments or comparable ethical standards.

## Supporting information


Figure S1

Table S1

## Data Availability

The datasets created and/or processed in this work may be found at the Surveillance, Epidemiology, and End Results Program repository, which can be found at https://seer.cancer.gov/data/index.html.
