# Supplementary figures and images for: Impact of prior cancer history on survival of patients with hypopharyngeal cancer
Source: Cancer Med. 2022 Sep 4;12(3):2929–36. doi: 10.1002/cam4.5208 (PMC9939181; doi:10.1002/cam4.5208)

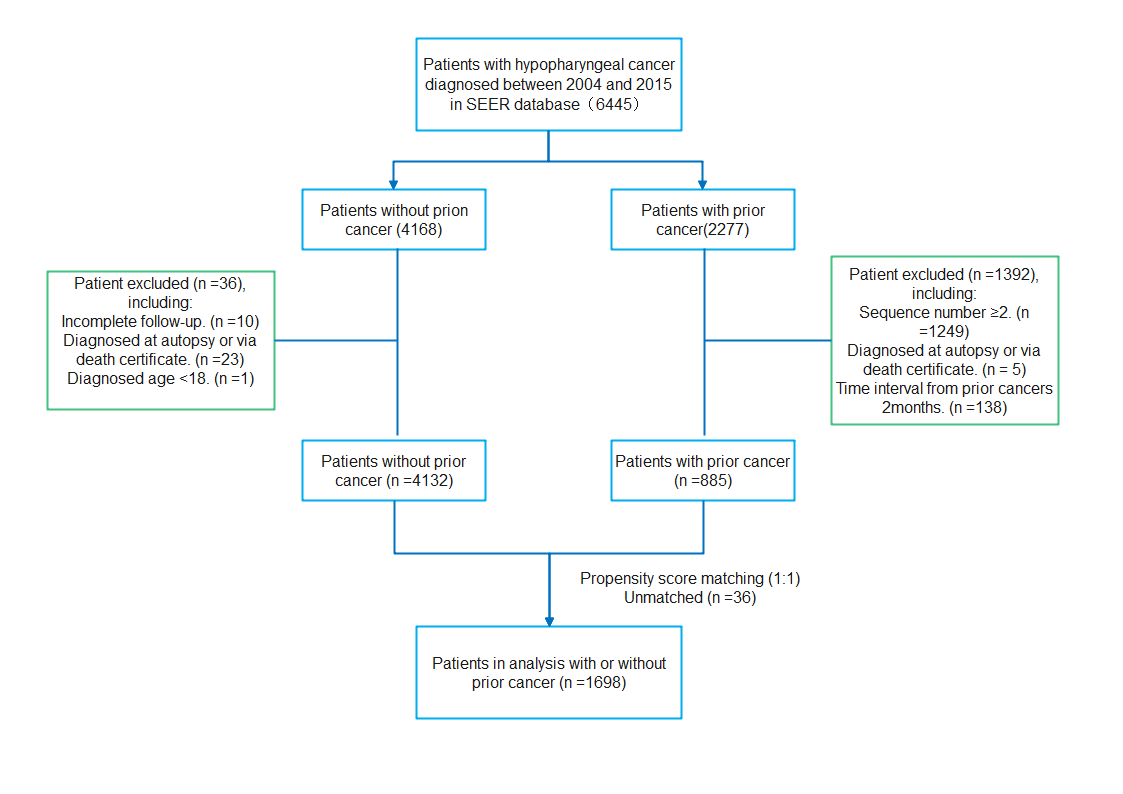

Supplement: Supplementary file 1 — Figure S1 [file CAM4-12-2929-s002.tiff]
