# Supplementary material for: Impact of prior cancer history on survival of patients with hypopharyngeal cancer
Source: Cancer Med. 2022 Sep 4;12(3):2929–36. doi: 10.1002/cam4.5208 (PMC9939181; doi:10.1002/cam4.5208)
Supplement: Supplementary file 2 — Table S1 [file CAM4-12-2929-s001.docx]

**Table S1 Distributions of prior cancer types** **for the 36 patients excluded in the matched dataset.**

| Prior cancer |  | Number | Proportion(%) | |  |
| --- | --- | --- | --- | --- | --- |
| Prostate | | 9 | | 25.0 | |
| Lung | | 5 | | 13.9 | |
| Glottis | | 4 | | 11.1 | |
| Breast | | 3 | | 8.3 | |
| Supraglottis | | 3 | | 8.3 | |
| Bladder | | 2 | | 5.6 | |
| Colon | | 2 | | 5.6 | |
| Tonsil | | 2 | | 5.6 | |
| throid | | 1 | | 2.8 | |
| stomach | | 1 | | 2.8 | |
| brain | | 1 | | 2.8 | |
| bone | | 1 | | 2.8 | |
| Rectum | | 1 | | 2.8 | |
| Tongue | | 1 | | 2.8 | |

**Table S2 Baseline characteristics of patients with hypopharyngeal cancer prior to prostate cancer.**

| Characteristic |  | Original data set | | | Matched data set | | |
| --- | --- | --- | --- | --- | --- | --- | --- |
|  |  | No prior cancer(N=849) | prior cancer(N=206) | P | No prior cancer(N=202) | prior cancer(N=202) | P |
| Age | | 263 (31.0)  586 (69.0)  432 (50.9)  417 (49.1)  674 (79.4)  114 (13.4)  61 (7.2)  191 (22.5)  658 (77.5)  172 (20.3)  362 (42.6)  279 (32.9)  36 (4.2)  40 (4.7)  307 (36.2)  284 (33.5)  14 (1.6)  204 (24.0)  118 (13.9)  651 (76.7)  80 (9.4)  605 (71.3)  244 (28.7)  417 (49.1)  432 (50.9)  305 (35.9)  544 (64.1) | 33 (16.0)  173 (84.0)  104 (50.5)  102 (49.5)  147 (71.4)  49 (23.8)  10 (4.9)  0 (0.0)  206 (100.0)  29 (14.1)  115 (55.8)  53 (25.7)  9 (4.4)  10 (4.9)  70 (34.0)  72 (35.0)  2 (1.0)  52 (25.2)  30 (14.6)  151 (73.3)  25 (12.1)  177 (85.9)  29 (14.1)  85 (41.3)  121 (58.7)  45 (21.8)  161 (78.2) | <0.001 | 33 (16.3)  169 (83.7)  99 (49.0)  103 (51.0)  159 (78.7)  26 (12.9)  17 (8.4)  0 (0.0)  202 (100.0)  31 (15.3)  117 (57.9)  47 (23.3)  7 (3.5)  9 (4.5)  71 (35.1)  72 (35.6)  3 (1.5)  47 (23.3)  19 (9.4)  169 (83.7)  14 (6.9)  177 (87.6)  25 (12.4)  83 (41.1)  119 (58.9)  48 (23.8)  154 (76.2) | 33 (16.3)  169 (83.7)  100 (49.5)  102 (50.5)  147 (72.8)  48 (23.8)  7 (3.5)  0 (0.0)  202 (100.0)  29 (14.4)  113 (55.9)  51 (25.2)  9 (4.5)  10 (5.0)  69 (34.2)  71 (35.1)  1 (0.5)  51 (25.2)  30 (14.9)  150 (74.3)  22 (10.9)  173 (85.6)  29 (14.4)  83 (41.1)  119 (58.9)  44 (21.8)  158 (78.2) | 1 |
| ＜65 | |  |  |  |  |  |  |
| ≥65 | |  |  |  |  |  |  |
| Year of diagnose | |  |  | 0.98 |  |  | 1 |
| 2004-2009 | |  |  |  |  |  |  |
| 2010-2015 | |  |  |  |  |  |  |
| Race | |  |  | 0.001 |  |  | 0.087 |
| White | |  |  |  |  |  |  |
| Black | |  |  |  |  |  |  |
| Others/Unknown | |  |  |  |  |  |  |
| Gender | |  |  | <0.001 |  |  | 0.908 |
| Female | |  |  |  |  |  |  |
| Male | |  |  |  |  |  |  |
| Marital status | |  |  | 0.006 |  |  | 0.87 |
| Single | |  |  |  |  |  |  |
| Married | |  |  |  |  |  |  |
| Other status | |  |  |  |  |  |  |
| Unknown | |  |  |  |  |  |  |
| Grade | |  |  | 0.919 |  |  | 0.068 |
| Grade Ⅰ | |  |  |  |  |  |  |
| Grade Ⅱ | |  |  |  |  |  |  |
| Grade Ⅲ | |  |  |  |  |  |  |
| Grade IⅤ | |  |  |  |  |  |  |
| Unknown | |  |  |  |  |  |  |
| AJCC | |  |  | 0.466 |  |  | 0.689 |
| Ⅰ-Ⅱ | |  |  |  |  |  |  |
| Ⅲ-Ⅳ | |  |  |  |  |  |  |
| Unknown | |  |  |  |  |  |  |
| Surgery | |  |  | <0.001 |  |  | 0.661 |
| No/unknown | |  |  |  |  |  |  |
| Yes | |  |  |  |  |  |  |
| Chemotherapy | |  |  | 0.052 |  |  | 1 |
| No/unknown | |  |  |  |  |  |  |
| Yes | |  |  |  |  |  |  |
| Radiotherapy | |  |  | <0.001 |  |  | 0.722 |
| No/unknown | |  |  |  |  |  |  |
| Yes | |  |  |  |  |  |  |

**Table S3 Baseline characteristics of patients with hypopharyngeal cancer prior to head and neck cancer.**

| Characteristic |  | Original data set | | | Matched data set | | |
| --- | --- | --- | --- | --- | --- | --- | --- |
|  |  | No prior cancer(N=849) | prior cancer(N=176) | P | No prior cancer(N=165) | prior cancer(N=165) | P |
| Age | | 263 (31.0)  586 (69.0)  432 (50.9)  417 (49.1)  674 (79.4)  114 (13.4)  61 (7.2)  191 (22.5)  658 (77.5)  172 (20.3)  362 (42.6)  279 (32.9)  36 (4.2)  40 (4.7)  307 (36.2)  284 (33.5)  14 (1.6)  204 (24.0)  118 (13.9)  651 (76.7)  80 (9.4)  605 (71.3)  244 (28.7)  417 (49.1)  432 (50.9)  305 (35.9)  544 (64.1) | 82 (46.6)  94 (53.4)  73 (41.5)  103 (58.5)  137 (77.8)  30 (17.0)  9 (5.1)  40 (22.7)  136 (77.3)  39 (22.2)  86 (48.9)  43 (24.4)  8 (4.5)  7 (4.0)  80 (45.5)  56 (31.8)  1 (0.6)  32 (18.2)  46 (26.1)  91 (51.7)  39 (22.2)  84 (47.7)  92 (52.3)  124 (70.5)  52 (29.5)  112 (63.6)  64 (36.4) | <0.001 | 80 (48.5)  85 (51.5)  55 (33.3)  110 (66.7)  122 (73.9)  34 (20.6)  9 (5.5)  42 (25.5)  123 (74.5)  25 (15.2)  88 (53.3)  47 (28.5)  5 (3.0)  8 (4.8)  82 (49.7)  44 (26.7)  1 (0.6)  30 (18.2)  38 (23.0)  109 (66.1)  18 (10.9)  114 (69.1)  51 (30.9)  99 (60.0)  66 (40.0)  72 (43.6)  93 (56.4) | 78 (47.3)  87 (52.7)  58 (35.2)  107 (64.8)  131 (79.4)  23 (13.9)  11 (6.7)  36 (21.8)  129 (78.2)  21 (12.7)  89 (53.9)  47 (28.5)  8 (4.8)  13 (7.9)  67 (40.6)  51 (30.9)  1 (0.6)  33 (20.0)  41 (24.8)  90 (54.5)  34 (20.6)  112 (67.9)  53 (32.1)  100 (60.6)  65 (39.4)  75 (45.5)  90 (54.5) | 0.912 |
| ＜65 | |  |  |  |  |  |  |
| ≥65 | |  |  |  |  |  |  |
| Year of diagnose | |  |  | 0.029 |  |  | 0.817 |
| 2004-2009 | |  |  |  |  |  |  |
| 2010-2015 | |  |  |  |  |  |  |
| Race | |  |  | 0.314 |  |  | 0.267 |
| White | |  |  |  |  |  |  |
| Black | |  |  |  |  |  |  |
| Others/Unknown | |  |  |  |  |  |  |
| Gender | |  |  | 0.345 |  |  | 0.517 |
| Female | |  |  |  |  |  |  |
| Male | |  |  |  |  |  |  |
| Marital status | |  |  | 0.181 |  |  | 0.790 |
| Single | |  |  |  |  |  |  |
| Married | |  |  |  |  |  |  |
| Other status | |  |  |  |  |  |  |
| Unknown | |  |  |  |  |  |  |
| Grade | |  |  | 0.138 |  |  | 0.500 |
| Grade Ⅰ | |  |  |  |  |  |  |
| Grade Ⅱ | |  |  |  |  |  |  |
| Grade Ⅲ | |  |  |  |  |  |  |
| Grade IⅤ | |  |  |  |  |  |  |
| Unknown | |  |  |  |  |  |  |
| AJCC | |  |  | <0.001 |  |  | 0.334 |
| Ⅰ-Ⅱ | |  |  |  |  |  |  |
| Ⅲ-Ⅳ | |  |  |  |  |  |  |
| Unknown | |  |  |  |  |  |  |
| Surgery | |  |  | <0.001 |  |  | 0.906 |
| No/unknown | |  |  |  |  |  |  |
| Yes | |  |  |  |  |  |  |
| Chemotherapy | |  |  | <0.001 |  |  | 1 |
| No/unknown | |  |  |  |  |  |  |
| Yes | |  |  |  |  |  |  |
| Radiotherapy | |  |  | <0.001 |  |  | 0.825 |
| No/unknown | |  |  |  |  |  |  |
| Yes | |  |  |  |  |  |  |

**Table S4 Baseline characteristics of patients with hypopharyngeal cancer prior to lung cancer.**

| Characteristic |  | Original data set | | | Matched data set | | |
| --- | --- | --- | --- | --- | --- | --- | --- |
|  |  | No prior cancer(N=849) | prior cancer(N=63) | P | No prior cancer(N=63) | prior cancer(N=63) | P |
| Age | | 263 (31.0)  586 (69.0)  432 (50.9)  417 (49.1)  674 (79.4)  114 (13.4)  61 (7.2)  191 (22.5)  658 (77.5)  172 (20.3)  362 (42.6)  279 (32.9)  36 (4.2)  40 (4.7)  307 (36.2)  284 (33.5)  14 (1.6)  204 (24.0)  118 (13.9)  651 (76.7)  80 (9.4)  605 (71.3)  244 (28.7)  417 (49.1)  432 (50.9)  305 (35.9)  544 (64.1) | 16 (25.4)  47 (74.6)  24 (38.1)  39 (61.9)  52 (82.5)  7 (11.1)  4 (6.3)  18 (28.6)  45 (71.4)  6 (9.5)  32 (50.8)  22 (34.9)  3 (4.8)  2 (3.2)  20 (31.7)  19 (30.2)  0 (0.0)  22 (34.9)  11 (17.5)  41 (65.1)  11 (17.5)  54 (85.7)  9 (14.3)  39 (61.9)  24 (38.1)  16 (25.4)  47 (74.6) | 0.432 | 16 (25.4)  47 (74.6)  29 (46.0)  34 (54.0)  52 (82.5)  8 (12.7)  3 (4.8)  20 (31.7)  43 (68.3)  10 (15.9)  34 (54.0)  16 (25.4)  3 (4.8)  5 (7.9)  18 (28.7)  20 (31.7)  20 (31.7)  0(0.0)  8 (12.7)  47 (74.6)  8 (12.7)  55 (87.3)  8 (12.7)  38 (60.3)  25 (39.7)  12 (19.0)  51 (81.0) | 16 (25.4)  47 (74.6)  24 (38.1)  39 (61.9)  52 (82.5)  7 (11.1)  4 (6.3)  18 (28.6)  45 (71.4)  6 (9.5)  32 (50.8)  22 (34.9)  3 (4.8)  2 (3.2)  20 (31.7)  19 (30.2)  22 (34.9)  0(0.0)  11 (17.5)  41 (65.1)  11 (17.5)  54 (85.7)  9 (14.3)  39 (61.9)  24 (38.1)  16 (25.4)  47 (74.6) | 1 |
| ＜65 | |  |  |  |  |  |  |
| ≥65 | |  |  |  |  |  |  |
| Year of diagnose | |  |  | 0.068 |  |  | 0.470 |
| 2004-2009 | |  |  |  |  |  |  |
| 2010-2015 | |  |  |  |  |  |  |
| Race | |  |  | 0.832 |  |  | 0.901 |
| White | |  |  |  |  |  |  |
| Black | |  |  |  |  |  |  |
| Others/Unknown | |  |  |  |  |  |  |
| Gender | |  |  | 0.341 |  |  | 0.846 |
| Female | |  |  |  |  |  |  |
| Male | |  |  |  |  |  |  |
| Marital status | |  |  | 0.214 |  |  | 0.571 |
| Single | |  |  |  |  |  |  |
| Married | |  |  |  |  |  |  |
| Other status | |  |  |  |  |  |  |
| Unknown | |  |  |  |  |  |  |
| Grade | |  |  | 0.324 |  |  | 0.680 |
| Grade Ⅰ | |  |  |  |  |  |  |
| Grade Ⅱ | |  |  |  |  |  |  |
| Grade Ⅲ | |  |  |  |  |  |  |
| Grade IⅤ | |  |  |  |  |  |  |
| Unknown | |  |  |  |  |  |  |
| AJCC | |  |  | 0.068 |  |  | 0.508 |
| Ⅰ-Ⅱ | |  |  |  |  |  |  |
| Ⅲ-Ⅳ | |  |  |  |  |  |  |
| Unknown | |  |  |  |  |  |  |
| Surgery | |  |  | 0.02 |  |  | 1 |
| No/unknown | |  |  |  |  |  |  |
| Yes | |  |  |  |  |  |  |
| Chemotherapy | |  |  | 0.068 |  |  | 1 |
| No/unknown | |  |  |  |  |  |  |
| Yes | |  |  |  |  |  |  |
| Radiotherapy | |  |  | 0.121 |  |  | 0.52 |
| No/unknown | |  |  |  |  |  |  |
| Yes | |  |  |  |  |  |  |

**Table S5 Baseline characteristics of patients with hypopharyngeal cancer prior to colorectal cancer.**

| Characteristic |  | Original data set | | | Matched data set | | |
| --- | --- | --- | --- | --- | --- | --- | --- |
|  |  | No prior cancer(N=849) | prior cancer(N=53) | P | No prior cancer(N=52) | prior cancer(N=52) | P |
| Age | | 263 (31.0)  586 (69.0)  432 (50.9)  417 (49.1)  674 (79.4)  114 (13.4)  61 (7.2)  191 (22.5)  658 (77.5)  172 (20.3)  362 (42.6)  279 (32.9)  36 (4.2)  40 (4.7)  307 (36.2)  284 (33.5)  14 (1.6)  204 (24.0)  118 (13.9)  651 (76.7)  80 (9.4)  605 (71.3)  244 (28.7)  417 (49.1)  432 (50.9)  305 (35.9)  544 (64.1) | 9 (17.0)  44 (83.0)  22 (41.5)  31 (58.5)  47 (88.7)  4 (7.5)  2 (3.8)  6 (11.3)  47 (88.7)  5 (9.4)  27 (50.9)  18 (34.0)  3 (5.7)  2 (3.8)  25 (47.2)  15 (28.3)  0 (0.0)  11 (20.8)  13 (24.5)  35 (66.0)  5 (9.4)  43 (81.1)  10 (18.9)  22 (41.5)  31 (58.5)  11 (20.8)  42 (79.2) | 0.046 | 5 (9.6)  47 (90.4)  20 (38.5)  32 (61.5)  46 (88.5)  4 (7.7)  2 (3.8)  6 (11.5)  46 (88.5)  6 (11.5)  26 (50.0)  20 (38.5)  0 (0.0)  3 (5.8)  23 (44.2)  16 (30.8)  0 (0.0)  10 (19.2)  9 (17.3)  37 (71.2)  6 (11.5)  45 (86.5)  7 (13.5)  27 (51.9)  25 (48.1)  15 (28.8)  37 (71.2) | 9 (17.3)  43 (82.7)  22 (42.3)  30 (57.7)  46 (88.5)  4 (7.7)  2 (3.8)  6 (11.5)  46 (88.5)  5 (9.6)  27 (51.9)  18 (34.6)  2 (3.8)  2 (3.8)  24 (46.2)  15 (28.8)  0 (0.0)  10 (19.2)  12 (23.1)  35 (67.3)  5 (9.6)  42 (80.8)  10 (19.2)  21 (40.4)  31 (59.6)  11 (21.2)  41 (78.8) | 0.389 |
| ＜65 | |  |  |  |  |  |  |
| ≥65 | |  |  |  |  |  |  |
| Year of diagnose | |  |  | 0.237 |  |  | 0.842 |
| 2004-2009 | |  |  |  |  |  |  |
| 2010-2015 | |  |  |  |  |  |  |
| Race | |  |  | 0.261 |  |  | 1 |
| White | |  |  |  |  |  |  |
| Black | |  |  |  |  |  |  |
| Others/Unknown | |  |  |  |  |  |  |
| Gender | |  |  | 0.082 |  |  | 1 |
| Female | |  |  |  |  |  |  |
| Male | |  |  |  |  |  |  |
| Marital status | |  |  | 0.258 |  |  | 0.529 |
| Single | |  |  |  |  |  |  |
| Married | |  |  |  |  |  |  |
| Other status | |  |  |  |  |  |  |
| Unknown | |  |  |  |  |  |  |
| Grade | |  |  | 0.519 |  |  | 0.096 |
| Grade Ⅰ | |  |  |  |  |  |  |
| Grade Ⅱ | |  |  |  |  |  |  |
| Grade Ⅲ | |  |  |  |  |  |  |
| Grade IⅤ | |  |  |  |  |  |  |
| Unknown | |  |  |  |  |  |  |
| AJCC | |  |  | 0.099 |  |  | 0.75 |
| Ⅰ-Ⅱ | |  |  |  |  |  |  |
| Ⅲ-Ⅳ | |  |  |  |  |  |  |
| Unknown | |  |  |  |  |  |  |
| Surgery | |  |  | 0.164 |  |  | 0.596 |
| No/unknown | |  |  |  |  |  |  |
| Yes | |  |  |  |  |  |  |
| Chemotherapy | |  |  | 0.351 |  |  | 0.325 |
| No/unknown | |  |  |  |  |  |  |
| Yes | |  |  |  |  |  |  |
| Radiotherapy | |  |  | 0.036 |  |  | 0.497 |
| No/unknown | |  |  |  |  |  |  |
| Yes | |  |  |  |  |  |  |

**Table S6 Baseline characteristics of patients with hypopharyngeal cancer of stage I&II.**

| Characteristic |  | Original data set | | | Matched data set | | |
| --- | --- | --- | --- | --- | --- | --- | --- |
|  |  | No prior cancer(N=449) | prior cancer(N=179) | P | No prior cancer(N=148) | prior cancer(N=148) | P |
| Age | | 231 (51.4)  218 (48.6)  200 (44.5)  249 (55.5)  369 (82.2)  52 (11.6)  28 (6.2)  128 (28.5)  321 (71.5)  71 (15.8)  253 (56.3)  111 (24.7)  14 (3.1)  18 (4.0)  177 (39.4)  130 (29.0)  9 (2.0)  115 (25.6)  337 (75.1)  112 (24.9)  258 (57.5)  191 (42.5)  108 (24.1)  341 (75.9) | 62 (34.6)  117 (65.4)  64 (35.8)  115 (64.2)  150 (83.8)  18 (10.1)  11 (6.1)  43 (24.0)  136 (76.0)  28 (15.6)  95 (53.1)  47 (26.3)  9 (5.0)  11 (6.1)  90 (50.3)  45 (25.1)  2 (1.1)  31 (17.3)  99 (55.3)  80 (44.7)  126 (70.4)  53 (29.6)  84 (46.9)  95 (53.1) | <0.001 | 59 (39.9)  89 (60.1)  46 (31.1)  102 (68.9)  122 (82.4)  16 (10.8)  10 (6.8)  36 (24.3)  112 (75.7)  15 (10.1)  91 (61.5)  40 (27.0)  2 (1.4)  4 (2.7)  66 (44.6)  46 (31.1)  1 (0.7)  31 (20.9)  90 (60.8)  58 (39.2)  98 (66.2)  50 (33.8)  49 (33.1)  99 (66.9) | 61 (41.2)  87 (58.8)  56 (37.8)  92 (62.2)  121 (81.8)  16 (10.8)  11 (7.4)  38 (25.7)  110 (74.3)  21 (14.2)  83 (56.1)  37 (25.0)  7 (4.7)  7 (4.7)  69 (46.6)  40 (27.0)  2 (1.4)  30 (20.3)  94 (63.5)  54 (36.5)  97 (65.5)  51 (34.5)  55 (37.2)  93 (62.8) | 0.906 |
| ＜65 | |  |  |  |  |  |  |
| ≥65 | |  |  |  |  |  |  |
| Year of diagnose | |  |  | 0.054 |  |  | 0.271 |
| 2004-2009 | |  |  |  |  |  |  |
| 2010-2015 | |  |  |  |  |  |  |
| Race | |  |  | 0.857 |  |  | 0.974 |
| White | |  |  |  |  |  |  |
| Black | |  |  |  |  |  |  |
| Others/Unknown | |  |  |  |  |  |  |
| Gender | |  |  | 0.298 |  |  | 0.893 |
| Female | |  |  |  |  |  |  |
| Male | |  |  |  |  |  |  |
| Marital status | |  |  | 0.649 |  |  | 0.234 |
| Single | |  |  |  |  |  |  |
| Married | |  |  |  |  |  |  |
| Other status | |  |  |  |  |  |  |
| Unknown | |  |  |  |  |  |  |
| Grade | |  |  | 0.043 |  |  | 0.799 |
| Grade Ⅰ | |  |  |  |  |  |  |
| Grade Ⅱ | |  |  |  |  |  |  |
| Grade Ⅲ | |  |  |  |  |  |  |
| Grade IⅤ | |  |  |  |  |  |  |
| Unknown | |  |  |  |  |  |  |
| Surgery | |  |  | <0.001 |  |  | 0.719 |
| No/unknown | |  |  |  |  |  |  |
| Yes | |  |  |  |  |  |  |
| Chemotherapy | |  |  | 0.004 |  |  | 1 |
| No/unknown | |  |  |  |  |  |  |
| Yes | |  |  |  |  |  |  |
| Radiotherapy | |  |  | <0.001 |  |  | 0.543 |
| No/unknown | |  |  |  |  |  |  |
| Yes | |  |  |  |  |  |  |

**Table S7 Baseline characteristics of patients with hypopharyngeal cancer of stage III&IV.**

| Characteristic |  | Original data set | | | Matched data set | | |
| --- | --- | --- | --- | --- | --- | --- | --- |
|  |  | No prior cancer(N=3278) | prior cancer(N=557) | P | No prior cancer(N=535) | prior cancer(N=535) | P |
| Age | | 1599 (48.8)  1679 (51.2)  1918 (58.5)  1360 (41.5)  2441 (74.5)  610 (18.6)  227 (6.9)  551 (16.8)  2727 (83.2)  864 (26.4)  1385 (42.3)  892 (27.2)  137 (4.2)  111 (3.4)  1230 (37.5)  1084 (33.1)  46 (1.4)  807 (24.6)  2760 (84.2)  518 (15.8)  914 (27.9)  2364 (72.1)  720 (22.0)  2558 (78.0) | 248 (44.5)  309 (55.5)  167 (30.0)  390 (70.0)  446 (80.1)  85 (15.3)  26 (4.7)  93 (16.7)  464 (83.3)  80 (14.4)  287 (51.5)  162 (29.1)  28 (5.0)  28 (5.0)  204 (36.6)  200 (35.9)  2 (0.4)  123 (22.1)  412 (74.0)  145 (26.0)  262 (47.0)  295 (53.0)  185 (33.2)  372 (66.8) | 0.07 | 258 (48.2)  277 (51.8)  145 (27.1)  390 (72.9)  438 (81.9)  71 (13.3)  26 (4.9)  88 (16.4)  447 (83.6)  90 (16.8)  269 (50.3)  154 (28.8)  22 (4.1)  12 (2.2)  193 (36.1)  191 (35.7)  5 (0.9)  134 (25.0)  426 (79.6)  109 (20.4)  223 (41.7)  312 (58.3)  157 (29.3)  378 (70.7) | 244 (45.6)  291 (54.4)  167 (31.2)  368 (68.8)  425 (79.4)  84 (15.7)  26 (4.9)  89 (16.6)  446 (83.4)  79 (14.8)  274 (51.2)  157 (29.3)  25 (4.7)  23 (4.3)  192 (35.9)  195 (36.4)  2 (0.4)  123 (23.0)  412 (77.0)  123 (23.0)  241 (45.0)  294 (55.0)  167 (31.2)  368 (68.8) | 0.426 |
| ＜65 | |  |  |  |  |  |  |
| ≥65 | |  |  |  |  |  |  |
| Year of diagnose | |  |  | <0.001 |  |  | 0.158 |
| 2004-2009 | |  |  |  |  |  |  |
| 2010-2015 | |  |  |  |  |  |  |
| Race | |  |  | 0.014 |  |  | 0.526 |
| White | |  |  |  |  |  |  |
| Black | |  |  |  |  |  |  |
| Others/Unknown | |  |  |  |  |  |  |
| Gender | |  |  | 0.997 |  |  | 1 |
| Female | |  |  |  |  |  |  |
| Male | |  |  |  |  |  |  |
| Marital status | |  |  | <0.001 |  |  | 0.805 |
| Single | |  |  |  |  |  |  |
| Married | |  |  |  |  |  |  |
| Other status | |  |  |  |  |  |  |
| Unknown | |  |  |  |  |  |  |
| Grade | |  |  | 0.037 |  |  | 0.262 |
| Grade Ⅰ | |  |  |  |  |  |  |
| Grade Ⅱ | |  |  |  |  |  |  |
| Grade Ⅲ | |  |  |  |  |  |  |
| Grade IⅤ | |  |  |  |  |  |  |
| Unknown | |  |  |  |  |  |  |
| Surgery | |  |  | <0.001 |  |  | 0.335 |
| No/unknown | |  |  |  |  |  |  |
| Yes | |  |  |  |  |  |  |
| Chemotherapy | |  |  | <0.001 |  |  | 0.294 |
| No/unknown | |  |  |  |  |  |  |
| Yes | |  |  |  |  |  |  |
| Radiotherapy | |  |  | <0.001 |  |  | 0.549 |
| No/unknown | |  |  |  |  |  |  |
| Yes | |  |  |  |  |  |  |

**Table S8 Baseline characteristics of patients with hypopharyngeal cancer of age <65 years.**

| Characteristic |  | Original data set | | | Matched data set | | |
| --- | --- | --- | --- | --- | --- | --- | --- |
|  |  | No prior cancer(N=2322) | prior cancer(N=277) | P | No prior cancer(N=256) | prior cancer(N=256) | P |
| Year of diagnose | | 1170 (50.4)  1152 (49.6)  1708 (73.6)  500 (21.5)  114 (4.9)  402 (17.3)  1920 (82.7)  766 (33.0)  927 (39.9)  521 (22.4)  108 (4.7)  91 (3.9)  901 (38.8)  729 (31.4)  28 (1.2)  573 (24.7)  200 (8.6)  1918 (82.6)  204 (8.8)  1912 (82.3)  410 (17.7)  644 (27.7)  1678 (72.3)  500 (21.5)  1822 (78.5) | 119 (43.0)  158 (57.0)  216 (78.0)  50 (18.1)  11 (4.0)  59 (21.3)  218 (78.7)  70 (25.3)  122 (44.0)  65 (23.5)  20 (7.2)  10 (3.6)  119 (43.0)  88 (31.8)  3 (1.1)  57 (20.6)  64 (23.1)  167 (60.3)  46 (16.6)  177 (63.9)  100 (36.1)  140 (50.5)  137 (49.5)  104 (37.5)  173 (62.5) | 0.023 | 125 (48.8)  131 (51.2)  195 (76.2)  46 (18.0)  15 (5.9)  49 (19.1)  207 (80.9)  60 (23.4)  115 (44.9)  68 (26.6)  13 (5.1)  9 (3.5)  112 (43.8)  80 (31.2)  1 (0.4)  54 (21.1)  46 (18.0)  174 (68.0)  36 (14.1)  169 (66.0)  87 (34.0)  116 (45.3)  140 (54.7)  82 (32.0)  174 (68.0) | 114 (44.5)  142 (55.5)  197 (77.0)  49 (19.1)  10 (3.9)  56 (21.9)  200 (78.1)  68 (26.6)  111 (43.4)  61 (23.8)  16 (6.2)  8 (3.1)  107 (41.8)  82 (32.0)  3 (1.2)  56 (21.9)  53 (20.7)  157 (61.3)  46 (18.0)  177 (69.1)  79 (30.9)  119 (46.5)  137 (53.5)  84 (32.8)  172 (67.2) | 0.376 |
| 2004-2009 | |  |  |  |  |  |  |
| 2010-2015 | |  |  |  |  |  |  |
| Race | |  |  | 0.283 |  |  | 0.576 |
| White | |  |  |  |  |  |  |
| Black | |  |  |  |  |  |  |
| Others/Unknown | |  |  |  |  |  |  |
| Gender | |  |  | 0.119 |  |  | 0.511 |
| Female | |  |  |  |  |  |  |
| Male | |  |  |  |  |  |  |
| Marital status | |  |  | 0.029 |  |  | 0.738 |
| Single | |  |  |  |  |  |  |
| Married | |  |  |  |  |  |  |
| Other status | |  |  |  |  |  |  |
| Unknown | |  |  |  |  |  |  |
| Grade | |  |  | 0.573 |  |  | 0.872 |
| Grade Ⅰ | |  |  |  |  |  |  |
| Grade Ⅱ | |  |  |  |  |  |  |
| Grade Ⅲ | |  |  |  |  |  |  |
| Grade IⅤ | |  |  |  |  |  |  |
| Unknown | |  |  |  |  |  |  |
| AJCC | |  |  | <0.001 |  |  | 0.274 |
| Ⅰ-Ⅱ | |  |  |  |  |  |  |
| Ⅲ-Ⅳ | |  |  |  |  |  |  |
| Unknown | |  |  |  |  |  |  |
| Surgery | |  |  | <0.001 |  |  | 0.509 |
| No/unknown | |  |  |  |  |  |  |
| Yes | |  |  |  |  |  |  |
| Chemotherapy | |  |  | <0.001 |  |  | 0.859 |
| No/unknown | |  |  |  |  |  |  |
| Yes | |  |  |  |  |  |  |
| Radiotherapy | |  |  | <0.001 |  |  | 0.925 |
| No/unknown | |  |  |  |  |  |  |
| Yes | |  |  |  |  |  |  |

**Table S9 Baseline characteristics of patients with hypopharyngeal cancer of age ≥65 years.**

| Characteristic |  | Original data set | | | Matched data set | | |
| --- | --- | --- | --- | --- | --- | --- | --- |
|  |  | No prior cancer(N=1810) | prior cancer(N=608) | P | No prior cancer(N=571) | prior cancer(N=571) | P |
| Year of diagnose | | 913 (50.4)  897 (49.6)  1428 (78.9)  218 (12.0)  164 (9.1)  379 (20.9)  1431 (79.1)  254 (14.0)  881 (48.7)  594 (32.8)  81 (4.5)  57 (3.1)  643 (35.5)  576 (31.8)  32 (1.8)  502 (27.7)  249 (13.8)  1360 (75.1)  201 (11.1)  1541 (85.1)  269 (14.9)  794 (43.9)  1016 (56.1)  543 (30.0)  1267 (70.0) | 274 (45.1)  334 (54.9)  499 (82.1)  72 (11.8)  37 (6.1)  119 (19.6)  489 (80.4)  65 (10.7)  335 (55.1)  182 (29.9)  26 (4.3)  37 (6.1)  233 (38.3)  196 (32.2)  4 (0.7)  138 (22.7)  115 (18.9)  390 (64.1)  103 (16.9)  447 (73.5)  161 (26.5)  345 (56.7)  263 (43.3)  241 (39.6)  367 (60.4) | 0.025 | 283 (49.6)  288 (50.4)  470 (82.3)  67 (11.7)  34 (6.0)  104 (18.2)  467 (81.8)  76 (13.3)  301 (52.7)  171 (29.9)  23 (4.0)  25 (4.4)  214 (37.5)  179 (31.3)  7 (1.2)  146 (25.6)  91 (15.9)  405 (70.9)  75 (13.1)  437 (76.5)  134 (23.5)  308 (53.9)  263 (46.1)  201 (35.2)  370 (64.8) | 266 (46.6)  305 (53.4)  464 (81.3)  70 (12.3)  37 (6.5)  115 (20.1)  456 (79.9)  64 (11.2)  315 (55.2)  169 (29.6)  23 (4.0)  29 (5.1)  210 (36.8)  191 (33.5)  4 (0.7)  137 (24.0)  101 (17.7)  371 (65.0)  99 (17.3)  447 (78.3)  124 (21.7)  309 (54.1)  262 (45.9)  208 (36.4)  363 (63.6) | 0.343 |
| 2004-2009 | |  |  |  |  |  |  |
| 2010-2015 | |  |  |  |  |  |  |
| Race | |  |  | 0.066 |  |  | 0.452 |
| White | |  |  |  |  |  |  |
| Black | |  |  |  |  |  |  |
| Others/Unknown | |  |  | 0.507 |  |  | 0.891 |
| Gender | |  |  |  |  |  |  |
| Female | |  |  |  |  |  |  |
| Male | |  |  |  |  |  |  |
| Marital status | |  |  | 0.032 |  |  | 0.452 |
| Single | |  |  |  |  |  |  |
| Married | |  |  |  |  |  |  |
| Other status | |  |  | 0.001 |  |  | 0.715 |
| Unknown | |  |  |  |  |  |  |
| Grade | |  |  |  |  |  |  |
| Grade Ⅰ | |  |  |  |  |  |  |
| Grade Ⅱ | |  |  |  |  |  |  |
| Grade Ⅲ | |  |  | <0.001 |  |  | 0.767 |
| Grade IⅤ | |  |  |  |  |  |  |
| Unknown | |  |  |  |  |  |  |
| AJCC | |  |  | <0.001 |  |  | 0.457 |
| Ⅰ-Ⅱ | |  |  |  |  |  |  |
| Ⅲ-Ⅳ | |  |  |  |  |  |  |
| Unknown | |  |  |  |  |  |  |
| Surgery | |  |  | <0.001 |  |  | 0.524 |
| No/unknown | |  |  |  |  |  |  |
| Yes | |  |  |  |  |  |  |
| Chemotherapy | |  |  |  |  |  |  |
| No/unknown | |  |  | <0.001 |  |  | 0.833 |
| Yes | |  |  |  |  |  |  |
| Radiotherapy | |  |  |  |  |  |  |
| No/unknown | |  |  | <0.001 |  |  | 0.711 |
| Yes | |  |  |  |  |  |  |

**Table S10 Baseline characteristics of patients with hypopharyngeal cancer with a prexious cancer history interval time <36 months.**

| Characteristic |  | Original data set | | | Matched data set | | |
| --- | --- | --- | --- | --- | --- | --- | --- |
|  |  | No prior cancer(N=4132) | prior cancer(N=267) | P | No prior cancer(N=257) | prior cancer(N=257) | P |
| Age | | 2083 (50.4)  2049 (49.6)  2322 (56.2)  1810 (43.8)  3136 (75.9)  718 (17.4)  278 (6.7)  781 (18.9)  3351 (81.1)  1020 (24.7)  1808 (43.8)  1115 (27.0)  189 (4.6)  148 (3.6)  1544 (37.4)  1305 (31.6)  60 (1.5)  1075 (26.0)  449 (10.9)  3278 (79.3)  405 (9.8)  3453 (83.6)  679 (16.4)  1438 (34.8)  2694 (65.2)  1043 (25.2)  3089 (74.8) | 136 (50.9)  131 (49.1)  102 (38.2)  165 (61.8)  211 (79.0)  37 (13.9)  19 (7.1)  48 (18.0)  219 (82.0)  43 (16.1)  128 (47.9)  81 (30.3)  15 (5.6)  10 (3.7)  97 (36.3)  87 (32.6)  1 (0.4)  72 (27.0)  51 (19.1)  159 (59.6)  57 (21.3)  208 (77.9)  59 (22.1)  135 (50.6)  132 (49.4)  92 (34.5)  175 (65.5) | 0.918 | 142 (54.8)  117 (45.2)  93 (35.9)  166 (64.1)  215 (83.0)  32 (12.4)  12 (4.6)  46 (17.8)  213 (82.2)  38 (14.7)  129 (49.8)  84 (32.4)  8 (3.1)  14 (5.4)  93 (35.9)  76 (29.3)  5 (1.9)  71 (27.4)  39 (15.1)  173 (66.8)  47 (18.1)  212 (81.9)  47 (18.1)  120 (46.3)  139 (53.7)  84 (32.4)  175 (67.6) | 135 (52.1)  124 (47.9)  102 (39.4)  157 (60.6)  205 (79.2)  35 (13.5)  19 (7.3)  44 (17.0)  215 (83.0)  43 (16.6)  125 (48.3)  78 (30.1)  13 (5.0)  10 (3.9)  96 (37.1)  86 (33.2)  1 (0.4)  66 (25.5)  48 (18.5)  157 (60.6)  54 (20.8)  205 (79.2)  54 (20.8)  129 (49.8)  130 (50.2)  86 (33.2)  173 (66.8) | 0.597 |
| ＜65 | |  |  |  |  |  |  |
| ≥65 | |  |  |  |  |  |  |
| Year of diagnose | |  |  | <0.001 |  |  | 0.468 |
| 2004-2009 | |  |  |  |  |  |  |
| 2010-2015 | |  |  |  |  |  |  |
| Race | |  |  | 0.335 |  |  | 0.377 |
| White | |  |  |  |  |  |  |
| Black | |  |  |  |  |  |  |
| Others/Unknown | |  |  |  |  |  |  |
| Gender | |  |  | 0.769 |  |  | 0.908 |
| Female | |  |  |  |  |  |  |
| Male | |  |  |  |  |  |  |
| Marital status | |  |  | 0.016 |  |  | 0.618 |
| Single | |  |  |  |  |  |  |
| Married | |  |  |  |  |  |  |
| Other status | |  |  |  |  |  |  |
| Unknown | |  |  |  |  |  |  |
| Grade | |  |  | 0.67 |  |  | 0.382 |
| Grade Ⅰ | |  |  |  |  |  |  |
| Grade Ⅱ | |  |  |  |  |  |  |
| Grade Ⅲ | |  |  |  |  |  |  |
| Grade IⅤ | |  |  |  |  |  |  |
| Unknown | |  |  |  |  |  |  |
| AJCC | |  |  | <0.001 |  |  | 0.334 |
| Ⅰ-Ⅱ | |  |  |  |  |  |  |
| Ⅲ-Ⅳ | |  |  |  |  |  |  |
| Unknown | |  |  |  |  |  |  |
| Surgery | |  |  | 0.021 |  |  | 0.506 |
| No/unknown | |  |  |  |  |  |  |
| Yes | |  |  |  |  |  |  |
| Chemotherapy | |  |  | <0.001 |  |  | 0.482 |
| No/unknown | |  |  |  |  |  |  |
| Yes | |  |  |  |  |  |  |
| Radiotherapy | |  |  | 0.001 |  |  | 0.925 |
| No/unknown | |  |  |  |  |  |  |
| Yes | |  |  |  |  |  |  |

**Table S11 Baseline characteristics of patients with hypopharyngeal cancer with a prexious cancer history interval time of more than 36 months and less than 60 months.**

| Characteristic |  | Original data set | | | Matched data set | | |
| --- | --- | --- | --- | --- | --- | --- | --- |
|  |  | No prior cancer(N=4132) | prior cancer(N=147) | P | No prior cancer(N=137) | prior cancer(N=137) | P |
| Age | | 2083 (50.4)  2049 (49.6)  2322 (56.2)  1810 (43.8)  3136 (75.9)  718 (17.4)  278 (6.7)  781 (18.9)  3351 (81.1)  1020 (24.7)  1808 (43.8)  1115 (27.0)  189 (4.6)  148 (3.6)  1544 (37.4)  1305 (31.6)  60 (1.5)  1075 (26.0)  449 (10.9)  3278 (79.3)  405 (9.8)  3453 (83.6)  679 (16.4)  1438 (34.8)  2694 (65.2)  1043 (25.2)  3089 (74.8) | 72 (49.0)  75 (51.0)  56 (38.1)  91 (61.9)  128 (87.1)  16 (10.9)  3 (2.0)  21 (14.3)  126 (85.7)  18 (12.2)  83 (56.5)  36 (24.5)  10 (6.8)  9 (6.1)  63 (42.9)  49 (33.3)  1 (0.7)  25 (17.0)  30 (20.4)  89 (60.5)  28 (19.0)  99 (67.3)  48 (32.7)  82 (55.8)  65 (44.2)  50 (34.0)  97 (66.0) | 0.918 | 71 (51.8)  66 (48.2)  53 (38.7)  84 (61.3)  122 (89.1)  13 (9.5)  2 (1.5)  19 (13.9)  118 (86.1)  15 (10.9)  86 (62.8)  30 (21.9)  6 (4.4)  3 (2.2)  60 (43.8)  44 (32.1)  0 (0.0)  30 (21.9)  26 (19.0)  87 (63.5)  24 (17.5)  101 (73.7)  36 (26.3)  76 (55.5)  61 (44.5)  47 (34.3)  90 (65.7) | 69 (50.4)  68 (49.6)  55 (40.1)  82 (59.9)  119 (86.9)  15 (10.9)  3 (2.2)  19 (13.9)  118 (86.1)  17 (12.4)  77 (56.2)  34 (24.8)  9 (6.6)  4 (2.9)  58 (42.3)  49 (35.8)  1 (0.7)  25 (18.2)  25 (18.2)  86 (62.8)  26 (19.0)  98 (71.5)  39 (28.5)  73 (53.3)  64 (46.7)  45 (32.8)  92 (67.2) | 0.904 |
| ＜65 | |  |  |  |  |  |  |
| ≥65 | |  |  |  |  |  |  |
| Year of diagnose | |  |  | <0.001 |  |  | 0.902 |
| 2004-2009 | |  |  |  |  |  |  |
| 2010-2015 | |  |  |  |  |  |  |
| Race | |  |  | 0.335 |  |  | 0.827 |
| White | |  |  |  |  |  |  |
| Black | |  |  |  |  |  |  |
| Others/Unknown | |  |  |  |  |  |  |
| Gender | |  |  | 0.125 |  |  | 1 |
| Female | |  |  |  |  |  |  |
| Male | |  |  |  |  |  |  |
| Marital status | |  |  | 0.045 |  |  | 0.689 |
| Single | |  |  |  |  |  |  |
| Married | |  |  |  |  |  |  |
| Other status | |  |  |  |  |  |  |
| Unknown | |  |  |  |  |  |  |
| Grade | |  |  | 0.23 |  |  | 0.754 |
| Grade Ⅰ | |  |  |  |  |  |  |
| Grade Ⅱ | |  |  |  |  |  |  |
| Grade Ⅲ | |  |  |  |  |  |  |
| Grade IⅤ | |  |  |  |  |  |  |
| Unknown | |  |  |  |  |  |  |
| AJCC | |  |  | 0.045 |  |  | 0.949 |
| Ⅰ-Ⅱ | |  |  |  |  |  |  |
| Ⅲ-Ⅳ | |  |  |  |  |  |  |
| Unknown | |  |  |  |  |  |  |
| Surgery | |  |  | <0.001 |  |  | 0.786 |
| No/unknown | |  |  |  |  |  |  |
| Yes | |  |  |  |  |  |  |
| Chemotherapy | |  |  | <0.001 |  |  | 0.808 |
| No/unknown | |  |  |  |  |  |  |
| Yes | |  |  |  |  |  |  |
| Radiotherapy | |  |  | <0.001 |  |  | 0.898 |
| No/unknown | |  |  |  |  |  |  |
| Yes | |  |  |  |  |  |  |

**Table S12 Baseline characteristics of patients with hypopharyngeal cancer with a prexious cancer history interval time >60 months.**

| Characteristic |  | Original data set | | | Matched data set | | |
| --- | --- | --- | --- | --- | --- | --- | --- |
|  |  | No prior cancer(N=849) | prior cancer(N=487) | P | No prior cancer(N=455) | prior cancer(N=455) | P |
| Age | | 2083 (50.4)  2049 (49.6)  2322 (56.2)  1810 (43.8)  3136 (75.9)  718 (17.4)  278 (6.7)  781 (18.9)  3351 (81.1)  1020 (24.7)  1808 (43.8)  1115 (27.0)  189 (4.6)  148 (3.6)  1544 (37.4)  1305 (31.6)  60 (1.5)  1075 (26.0)  449 (10.9)  3278 (79.3)  405 (9.8)  3453 (83.6)  679 (16.4)  1438 (34.8)  2694 (65.2)  1043 (25.2)  3089 (74.8) | 191 (39.2)  296 (60.8)  123 (25.3)  364 (74.7)  388 (79.7)  71 (14.6)  28 (5.7)  109 (22.4)  378 (77.6)  78 (16.0)  252 (51.7)  136 (27.9)  21 (4.3)  30 (6.2)  198 (40.7)  156 (32.0)  5 (1.0)  98 (20.1)  104 (21.4)  315 (64.7)  68 (14.0)  327 (67.1)  160 (32.9)  276 (56.7)  211 (43.3)  207 (42.5)  280 (57.5) | 0.458 | 184 (40.4)  271 (59.6)  109 (24.0)  346 (76.0)  366 (80.4)  61 (13.4)  28 (6.2)  103 (22.6)  352 (77.4)  85 (18.7)  221 (48.6)  136 (29.9)  13 (2.9)  20 (4.4)  194 (42.6)  145 (31.9)  6 (1.3)  90 (19.8)  86 (18.9)  322 (70.8)  47 (10.3)  343 (75.4)  112 (24.6)  239 (52.5)  216 (47.5)  157 (34.5)  298 (65.5) | 186 (40.9)  269 (59.1)  123 (27.0)  332 (73.0)  360 (79.1)  68 (14.9)  27 (5.9)  98 (21.5)  357 (78.5)  76 (16.7)  234 (51.4)  126 (27.7)  19 (4.2)  26 (5.7)  179 (39.3)  148 (32.5)  5 (1.1)  97 (21.3)  95 (20.9)  295 (64.8)  65 (14.3)  327 (71.9)  128 (28.1)  244 (53.6)  211 (46.4)  176 (38.7)  279 (61.3) | 0.946 |
| ＜65 | |  |  |  |  |  |  |
| ≥65 | |  |  |  |  |  |  |
| Year of diagnose | |  |  | 0.532 |  |  | 0.323 |
| 2004-2009 | |  |  |  |  |  |  |
| 2010-2015 | |  |  |  |  |  |  |
| Race | |  |  | <0.001 |  |  | 0.799 |
| White | |  |  |  |  |  |  |
| Black | |  |  |  |  |  |  |
| Others/Unknown | |  |  |  |  |  |  |
| Gender | |  |  | 0.225 |  |  | 0.749 |
| Female | |  |  |  |  |  |  |
| Male | |  |  |  |  |  |  |
| Marital status | |  |  | 0.145 |  |  | 0.497 |
| Single | |  |  |  |  |  |  |
| Married | |  |  |  |  |  |  |
| Other status | |  |  |  |  |  |  |
| Unknown | |  |  |  |  |  |  |
| Grade | |  |  | 0.087 |  |  | 0.778 |
| Grade Ⅰ | |  |  |  |  |  |  |
| Grade Ⅱ | |  |  |  |  |  |  |
| Grade Ⅲ | |  |  |  |  |  |  |
| Grade IⅤ | |  |  |  |  |  |  |
| Unknown | |  |  |  |  |  |  |
| AJCC | |  |  | 0.025 |  |  | 0.104 |
| Ⅰ-Ⅱ | |  |  |  |  |  |  |
| Ⅲ-Ⅳ | |  |  |  |  |  |  |
| Unknown | |  |  |  |  |  |  |
| Surgery | |  |  | <0.001 |  |  | 0.259 |
| No/unknown | |  |  |  |  |  |  |
| Yes | |  |  |  |  |  |  |
| Chemotherapy | |  |  | 0.025 |  |  | 0.790 |
| No/unknown | |  |  |  |  |  |  |
| Yes | |  |  |  |  |  |  |
| Radiotherapy | |  |  | <0.001 |  |  | 0.215 |
| No/unknown | |  |  |  |  |  |  |
| Yes | |  |  |  |  |  |  |
